# Whole-Genome Sequences of *Salmonella enterica* subsp. *enterica* Serovar Typhimurium Strains TT6675 and TT9097 Employed in the Isolation and Characterization of a Giant Phage Mutant Collection

**DOI:** 10.1128/genomeA.00857-17

**Published:** 2017-08-24

**Authors:** Han Ming Gan, Wilhelm Wei Han Eng, Melissa K. Barton, Lily E. Adams, Nurul Aisyah Samsudin, Austin J. Bartl, André O. Hudson, Michael A. Savka, Julie A. Thomas

**Affiliations:** a Centre for Integrative Ecology, School of Life and Environmental Sciences, Deakin University, Waurn Ponds, Victoria, Australia; b School of Science, Monash University Malaysia, Petaling Jaya, Selangor, Malaysia; c Genomics Facility, Tropical Medicine and Biology Platform, Monash University Malaysia, Petaling Jaya, Selangor, Malaysia; d Thomas H. Gosnell School of Life Sciences, Rochester Institute of Technology, Rochester, New York, USA

## Abstract

We report here the genome sequences of *Salmonella enterica* subsp. *enterica* serovar Typhimurium strains TT6675 and TT9097, which we utilize for genetic analyses of giant bacterial viruses. Our analyses identified several genetic variations between the two strains, most significantly confirming strain TT6675 as a serine suppressor and TT9097 as a nonsuppressor.

## GENOME ANNOUNCEMENT

The characterization of giant bacterial viruses (phages) with genomes >200 kb and complex virions are of interest to our research group. Recent studies have indicated that giant phages are abundant in the environment, infect a broad range of bacterial hosts, and have the potential for therapeutic applications (e.g., phage therapy). However, we have a limited understanding of the biology of giant phages due to the fact that many of their genes are functionally uncharacterized. To address this problem, we utilized two strains of *Salmonella enterica* subsp. *enterica* serovar Typhimurium LT2, TT6675 and TT9097, to successfully isolate an amber mutant collection for giant phage SPN3US (1). To undertake further studies of SPN3US, particularly omics analyses of infection, we needed to define the nucleotide sequences of these two strains.

Cells were cultured in nutrient medium, and genomic DNA was isolated using the GenElute bacterial genomic kit (Sigma-Aldrich, St. Louis, MO, USA). Sequencing libraries were prepared using the Nextera XT library prep kit (Illumina, San Diego, CA, USA) and tagged with unique dual-index barcodes. Sequencing was performed on the MiSeq desktop sequencer (Illumina) located at the Monash University Malaysia Genomics Facility. Paired-end reads were error-corrected and assembled *de novo* using SPAdes version 2.7 (2). The draft genomes were subsequently annotated via the NCBI Prokaryotic Genome Annotation Pipeline (3). Transfer RNA genes were manually verified using tRNA-Scan (4) and Aragorn (5).

The genome length of each strain is 4.86 Mb, with G+C contents of 52.23% and 52.27% for strains TT6675 and TT9097, respectively. Strain TT6675 was determined to encode 77 standard tRNAs, whereas strain TT9097 was determined to encode 78 standard tRNAs. This variation was due to the identification of a single nucleotide mutation in the anticodon (CGA-CTA) of the serine tRNA in TT6675 (serU of the type strain [6]), which produces a suppressor tRNA. This resolved the genetic basis for the suppressor and nonsuppressor phenotypes observed for TT6675 and TT9097, respectively. This is important for further work, as we can now incorporate accurate mutant peptides into mass spectral search libraries for analyses of mutant phage proteomes. Amber mutations in the histidinol-phosphate aminotransferase (*hisC*) and 2-isopropylmalate synthase (*leuA*) genes were identified in both strains, consistent with their reported phenotypes (J. Roth, personal communication). In addition, a 93.9-kb pSLT conjugative plasmid (7) was identified on a single contig in strain TT6675 (sequence no. MCGC01000009) and on five contigs in TT9097 (sequence no. MCGM0100112, MCGM01000114, MCGM01000149, and MCGM01000034). Components of the Tn*10* transposon were identified, although neither strain contains the reportedly inactive IS*10*-left transposase. TT6675 has only the IS*10*-right transposase gene, whereas TT9097 has *jemA*, *jemB*, *jemC*, *tetR*, *tetA*, *tetC*, *tetD*, and IS*10*-right transposase genes. The determination of the nucleotide sequences and variations between these two *S*. Typhimurium strains will be valuable for analyses of the molecular events during giant phage infection.

### Accession number(s).

Nucleotide sequences for the *S*. Typhimurium strains reported here have been deposited at DDBJ/EMBL/GenBank under the accession no. MCGL01000000 for TT6675 and MCGM01000000 for TT9097. The versions described in this paper are the first versions.

## References

[1] Thomas JA, Benítez Quintana AD, Bosch MA, Coll De Peña A, Aguilera E, Coulibaly A, Wu W, Osier MV, Hudson AO, Weintraub ST, Black LW 2016 Identification of essential genes in the *Salmonella* phage SPN3US reveals novel insights into giant phage head structure and assembly. J Virol 90:10284–10298. doi:10.1128/JVI.01492-16. 27605673 PMC5105663

[2] Bankevich A, Nurk S, Antipov D, Gurevich AA, Dvorkin M, Kulikov AS, Lesin VM, Nikolenko SI, Pham S, Prjibelski AD, Pyshkin AV, Sirotkin AV, Vyahhi N, Tesler G, Alekseyev MA, Pevzner PA 2012 SPAdes: a new genome assembly algorithm and its applications to single-cell sequencing. J Comput Biol 19:455–477. doi:10.1089/cmb.2012.0021. 22506599 PMC3342519

[3] Tatusova T, DiCuccio M, Badretdin A, Chetvernin V, Nawrocki EP, Zaslavsky L, Lomsadze A, Pruitt KD, Borodovsky M, Ostell J 2016 NCBI prokaryotic genome annotation pipeline. Nucleic Acids Res 44:6614–6624. doi:10.1093/nar/gkw569. 27342282 PMC5001611

[4] Lowe TM, Eddy SR 1997 TRNAscan-SE: a program for improved detection of transfer RNA genes in genomic sequence. Nucleic Acids Res 25:955–964. 9023104 10.1093/nar/25.5.955 PMC146525

[5] Laslett D, Canback B 2004 ARAGORN, a program to detect tRNA genes and tmRNA genes in nucleotide sequences. Nucleic Acids Res 32:11–16. doi:10.1093/nar/gkh152. 14704338 PMC373265

[6] McClelland M, Sanderson KE, Spieth J, Clifton SW, Latreille P, Courtney L, Porwollik S, Ali J, Dante M, Du F, Hou S, Layman D, Leonard S, Nguyen C, Scott K, Holmes A, Grewal N, Mulvaney E, Ryan E, Sun H, Florea L, Miller W, Stoneking T, Nhan M, Waterston R, Wilson RK 2001 Complete genome sequence of *Salmonella enterica* serovar Typhimurium LT2. Nature 413:852–856. 11677609 10.1038/35101614

[7] Ahmer BMM, Tran M, Heffron F 1999 The virulence plasmid of *Salmonella typhimurium* is self-transmissible. J Bacteriol 181:1364–1368. 9973370 10.1128/jb.181.4.1364-1368.1999 PMC93521

